# Policy and public communication methods among U.S. state prisons during the first year of the COVID-19 pandemic

**DOI:** 10.1186/s40352-022-00187-5

**Published:** 2022-09-01

**Authors:** Melissa J. Zielinski, Mariah Cowell, Chelsey E. Bull, Manasa Veluvolu, M. Forrest Behne, Kathryn Nowotny, Lauren Brinkley-Rubinstein

**Affiliations:** 1 Psychiatric Research Institute, University of Arkansas for Medical Sciences, Little Rock, AR USA; 2 Department of Psychological Science, University of Arkansas, Fayetteville, AR USA; 3 Department of Social Medicine, University of North Carolina at Chapel Hill, Chapel Hill, NC USA; 4 School of Social Work, University of North Carolina at Chapel Hill, Chapel Hill, NC USA; 5 Department of Sociology, University of Miami, Coral Gables, FL USA

**Keywords:** COVID-19, Prison, Policy, Visitation, Infection control

## Abstract

**Background:**

Throughout the first year of the COVID-19 pandemic, our research team monitored and documented policy changes in United States (U.S.) prison systems. Data sources included prison websites and official prison social media accounts. Over 2500 data sources relevant to the COVID-19 pandemic in U.S. prisons were located and summarized in to five different categories: 1) prevention, 2) case identification and intervention, 3) movement, 4) social communication and connection, and 5) programming, recreation, and privileges.

**Results:**

All state prison systems reportedly enacted multiple policies intended to limit the spread of COVID-19 during the pandemic. Document analysis revealed that the most commonly released policies were restrictions on social contacts and privileges, basic preventive measures (e.g., distribution of masks), and basic case identification measures (e.g., verbal screening and temperature checks). Utilization of social media for policy communication varied significantly across states, though relevant data was more often released on Facebook than Twitter.

**Conclusions:**

Together, our work provides foundational knowledge on the wide breadth of policies that were reportedly enacted in the first year of the pandemic that may be used as a base for quantitative work on policy effectiveness and examinations of implementation.

## Introduction

People who are incarcerated are at increased risk for COVID-19 acquisition (Kinner et al., 2020), and large outbreaks of COVID-19 have been documented in carceral settings around the world (Rapisarda et al., 2020a, b; Rapisarda & Byrne, 2020a, b, c). As of June 10, 2022, at least 592,974 people incarcerated in U.S. prison systems had tested positive for the virus, and at least 2896 had died (COVID Prison Project, 2022). There had also been 205,390 cases and 278 deaths among prison staff. The rate of infection has been estimated to be 5 times higher among people who are incarcerated compared to the U.S. general public (Saloner et al., 2020), with substantial variation across U.S. states (Lemasters et al., 2020). Hazardous environmental conditions amplify the risks of exposure for both incarcerated people and carceral staff (Gershon et al., 2007; Nijhawan, 2016), and the built environment of prison facilities—which are typically overcrowded—make common COVID-19 prevention strategies such as social distancing nearly impossible (Bick, 2007). Prisons also often hold people who have a high burden of chronic disease (Binswanger et al., 2009), putting them at risk of suffering more severely from COVID-19 infection.

Despite widespread advocacy for decarceration from prison stakeholders and public health experts based on expectations that COVID-19 would devastate these systems (Rich et al., 2020; Howell et al., 2020; Ransom & Feuer, 2020), U.S. state prison population reductions have ultimately been minimal and slow. Consequently, effective policy and policy implementation has been the best hope for infection control, with agencies such as the Centers for Disease Control and Prevention and the National Commission on Correctional Health Care offering guidance for correctional institutions throughout the pandemic. Policies that have been enacted to prevent COVID-19 infections in the community have informed prison policies, but arguably have a greater degree of collateral consequences when applied to prisons. For example, suspending in-person visitation as a method to increase social distancing deprives people fully of any in-person contact with their social support network.

### COVID-19 prison policy research

The nature of carceral systems makes it inherently challenging to assess their pandemic responses. Communication between people who are incarcerated and people who are not has long been inconvenient and financially burdensome. Distance communication methods such as phone and/or video calls are often available but costly and privacy-limited. Incoming and outgoing mail is checked and subject to facility restrictions (e.g., a maximum number of pages). Thus, even prior to the pandemic, getting timely and accurate information from incarcerated people was problematic. Moreover, “inmate grievance” procedures—official channels by which prison residents can formally address concerns such as living conditions or health care access—remain largely inaccessible to outside parties. Internal grievances are also impeded by factors such as difficulties with written and verbal expression, fears of retaliation, associated fees (e.g., for filing an appeal), and risk of punitive actions for appeals judged to be false (Calavita & Jenness, 2015). Virtually all departments of corrections (DOC) mandate that such disputes be investigated, resolved, and responded to within the department and are free from external oversight.

Accounts from carceral staff are generally limited as well. A confluence of factors— including but not limited to pressure from commanding officers, a historical animus towards whistleblowers, and the threat of reprisals for perceived disloyalty—create barriers to officers who may wish to report possible non-compliance among their colleagues (Dryburgh, 2009). Thus, publicly available documents freely shared by prison systems and those that become available through court records are the primary sources that can be readily leveraged by researchers.

To the authors’ knowledge, two previous studies have examined prison policy responses to date. The most comprehensive prior report was published by Novisky et al. (2020). This study described the strengths and deficiencies of institutional responses to the COVID-19 by U.S. prisons early in the pandemic using data gathered from a one-time web scraping completed in June 2020. The study reported the following as strengths of institutional responses as of that date: 1) the existence of at least some form of public-facing COVID-19 updates in all states; 2) that most states made efforts to post information about confirmed COVID-19 cases and testing; 3) the existence of efforts to offset visitation restrictions with expansion in other social communication methods, and 4) the existence of other preventative efforts focused on reducing disease transmission. However, Novisky et al. (2020) also noted deficiencies including: 1) that the testing and COVID-19 case data was incomplete and lacked transparency; 2) inconsistent access to and permissibility of personal protective equipment for both incarcerated people and staff, and 3) continued restrictions on products such as hand sanitizer that could have been used in mitigation efforts. The data reported within the study was in some cases policy-focused (e.g., reporting the percentage of states that had suspended visitation) and in other cases focused on information accessibility (e.g., reporting the number of states that were openly reporting their COVID-19 testing and infection rates rather than on what the states’ policies were regarding testing).

A second study, research by Dallaire et al. (2021), focused specifically on reporting policies that affected communication between people who are incarcerated and their family members (e.g., visitation, phone access, email access). Data used in the study were collected during a one-time web scraping done over the course of a week in May 2020. This study highlighted that in-person visitation was suspended by March 19, 2020 in all 50 states—a notably rapid and uniform policy change. By the time that data was collected, nearly all states had begun to offer some number of free phone calls and/or extra minutes. The addition of other policies intended to offset limitations on in-person visitation such as free video calls, free emails, and free postage/stamps was more variable.

Both of these prior studies offer an important window in to prison responses to the COVID-19 pandemic. However, there is a need for additional research that is more comprehensive in methods (i.e. moving beyond a single web-scraping) and in scope (i.e. examining a broader range of policies over a longer monitoring period, examining a broader range of informational release methods including social media). In this paper, we provide an updated policy analysis for prison systems in the United States 1 year into the pandemic.

### ﻿The current study

In this study we: 1) summarize the communication methods from U.S. state prison systems to the public about COVID-19 mitigation efforts and 2) describe the policy changes made in U.S. state prisons since the onset of the COVID-19 pandemic. We focused on the first year of the pandemic (January 2020 to December 2020) to capture the onset of policy change and place primary emphasis on policies that were in place prior to the onset of vaccine distribution efforts.

## Methods

### COVID Prison Project

The COVID Prison Project (CPP) was founded in March 2020, at the beginning of the COVID-19 pandemic in the U.S. The goal of CPP was to systematically aggregate data on COVID-19 infection and death rates among incarcerated people and staff in U.S. prisons and jails. To date, the CPP team has aggregated data from 53 prison systems (i.e., Federal Bureau of Prisons, Immigration and Customs Enforcement, Puerto Rico, and all 50 U.S. states) and over 50 of the largest U.S. jails.

Beginning in June 2020, the CPP leadership team began a collaborative effort to expand CPP’s scope to include tracking of COVID-19 policies in state prison systems. We focused on state prison systems because of the public and centralized nature of these agencies, which would make policy decisions and communication possible to track. The CPP Policy Arm subsequently tracked, stored, coded, and analyzed the policies directly related to COVID-19 and its collateral consequences (i.e., implications for programming, in-prison socialization, and legal rights) for prison systems in all 50 states dating back to January 2020 and spanning through December 2020.

### Procedure

#### Document retrieval

Data for our policy monitoring were collected from publicly-available sources on statewide prison policy including DOC websites, Facebook pages, and Twitter pages for all 50 U.S. states. Our data collection team, comprised of trained research assistants, collectively used manual web scraping to collect all documents and notifications concerning policy changes from these official DOC sources. Research assistants retrieved documents from one or more groups of 3–4 states at least once per week for the duration of the project. Policies from each data source were then classified using our policy codebook (see Policy codebook subsection) and entered in to a database that was designed to track policies of interest across prison systems. The web address for each source of information to be scraped was compiled on a shared document accessible to all of the data collection team members contributing to document retrieval so that the sources from which materials were to be pulled was standardized. As data sources were identified, they were downloaded and saved to a shared file storage system organized by state.

##### Data source inclusion and exclusion criteria.

Policies, social media posts, and other source documents that referenced practices or policies intended to mitigate COVID-19 and/or that were changed as a result of COVID-19 within the state DOC were downloaded for use in analyses. We also downloaded documents that visually depicted (non)implementation of policies (e.g., lack of masking in staff photos posted to social media). We did not include documents that were simply re-posts of guidance from state departments of health or other sources unless there was a reference to how the DOC was using the document(s).

#### Policy codebook

Our codebook outlined 52 policies to be monitored. These 52 policies are summarized in five superordinate policy categories here for conceptual clarity: 1) Prevention, 2) Case Identification and Intervention; 3) Movement 4) Social Communication and Connection; and 5) Programming, Recreation, and Privileges. Table 1 provides our operationalization of each superordinate policy category; please see Appendix 1 for our full policy codebook (i.e., the names and definitions of all 52 policies that we monitored).^1^

**Table 1:** Definitions of policy categories

Category	Definition
Prevention	Prevention policies included measures taken to preemptively mitigate the spread of COVID-19 through masking, reducing and suspending intakes, staff quarantining, distribution of hand sanitizer, and increased facility cleaning.
Case Identification and Intervention	Identification and intervention policies included policies related to COVID-19 screening, testing, medical care, and medical isolation.
Movement	Movement policies included restrictions placed on individuals who are incarcerated and their movement around facilities, such as social distancing and transfer restrictions.
Social Communication and Connection	Social policies included measures that impacted incarcerated persons’ ability to connect to their social networks such as visitation restrictions and expanded access to distance communication methods (e.g., phone or video calls, secure messaging, emails).
Programming, Recreation, and Privileges	Privileges include policies that focus on expansion and restriction around how individuals who are incarcerated are able to spend their time. This includes reductions or increases to recreation time, limited work release jobs, and programming reduced or suspended.

In our aggregate reporting of each policy, we also indicate 1) the policy type and form and 2) who the policy primarily affects (see Table 2 for operationalizations).

**Table 2:** Categories and definitions of policy classifications

Classification Category	Definition
**Policy Type**	
Public health	Policies that mirror broader public health measures or mandates
DOC-specific	Policies that address actions or needs that only apply to correctional agencies or systems
**Policy Form**	
Expand access	Policies that make something more available than it was previously, either by explicitly authorizing increased access or by reducing barriers to access
Reduce access	Policies that make something less available than it was previously, either by explicitly suspending or reducing access or by increasing barriers to access
Mandate change	Policies that create requirements for individuals or organizations
**Who Policy Primarily Affects**	
Resident	Policies that primarily target individual residents and/or resident behavior
Staff	Policies that primarily target staff and/or staff behavior
System	Policies that primarily target or facilitate institutional change

#### Data collection tool

The data collection tool directly mapped on to the list of policy categories and sub-codes. While we initially attempted to incorporate beginning and end dates for the policies into our data collection, we ultimately focused our results on whether each state has *ever* enacted each policy of interest.^2^

#### Data collection team structure

The research team consisted of a project coordinator and research assistants who were assigned to monitor all sources of information and download related documents for one or more groups of 3–4 states at least weekly (ex. AL, AK, AR, AZ). There were a total 13 groups, each of which was assigned one person to monitor. Each team member was responsible for one or more groups of four states; groups were established to create a manageable monitoring load given that, especially early in the pandemic, some states were releasing policies often.

Over the course of the project, 16 individuals contributed to document retrieval and data extraction. State groups were held constant and assignments were held constant unless a team member needed to rotate off of the project. This process was in place to promote increased familiarity with state communication systems and policies as the pandemic progressed. For continuity, the same RA who retrieved each piece of communication was responsible for coding each policy, press release, or social media post. Retrieved data sources were from each state’s DOC webpages and agency social media accounts (i.e., Twitter, Facebook).

### Analysis

Prior to analysis, the coding for all states was reviewed and checked for consistency with downloaded policy documents. Discrepancies were resolved by having an independent reviewer conduct a third check to determine the correct value. The research team calculated descriptive statistics summarizing the number and percentage of states releasing each of the policies that we monitored from the cleaned data. For the purposes of this report, the researchers only examined policies that were released in 2020. 

## Results

Over 2500 pieces of data consisting of information from DOC webpages and social media were obtained during the web scraping that was conducted for this study. The number of pieces of data obtained from each state ranged from 8 (South Dakota) to 164 (Washington), with the median number of items catalogued per state being 46.5 and mean being 50. Approximately three-quarters of our source data was from DOC webpages, while the remaining quarter was from social media.

### Policy communication

Of the 50 states, three states communicated through only one communication stream—a DOC website in all cases; this means that most states (94%) released information using at least one social media platform at least once during the pandemic. Facebook was most commonly utilized, with a sizeable majority of states (86%) releasing information via Facebook at least once during our monitoring period. Twitter was also used by most states (70%). Together, over half (58%) of institutions used all three forms of communication for disseminating information pertinent to COVID-19 policies and practices during our monitoring period (*n* = 29). Please see Appendix 2 for breakdown of communication platforms by each state.

#### Social media

More of our social media data came from Facebook than Twitter (18% versus 8% of total source data respectively). However, there was significant variability across states in terms of both frequency and content of social media usage for purposes related to the pandemic. For example, California, Arkansas, and Oklahoma used Twitter much more frequently than they used Facebook for relevant informational releases whereas 20 of the states that used Twitter had only five or fewer Twitter posts during the monitoring period that met our inclusion criteria.^3^ On the other hand, there were several states from which more policy-relevant data points were extracted from social media than from official DOC webpages. For example, Rhode Island DOC largely communicated via Facebook during the monitoring period.

The content of social media posts spanned the full range of the policy types that we report in the next section. Announcements related to social policies (e.g., suspension of visitation) and to programming policies (e.g., suspension of programs, updates about ongoing programs) were particularly common. Notably, there were occasions in which photos that accompanied social media posts seemed to contradict current policy (e.g., photos of residents gathered together in groups without masks worn or worn properly when distancing and/or masking policies were in place). This raises critical questions about policy implementation that were beyond the scope of the current study but that should be considered in future investigations.

### Policy content

Results of our document analysis are presented by our five major monitoring categories (i.e., prevention; case identification and intervention; movement; social connections; privileges) below. For each policy, we indicate the total number of state prison systems that released a policy and percentage out of the total number of states monitored (i.e., 50).

#### Prevention policies

While all policies coded in this study were in some way related to COVID-19 prevention, the policies that we included in this category were those intended to be implemented to preempt virus transmission. All mirrored public health interventions that were being taken in community settings such as masking and greater attention to sanitizing hands and surfaces (see Table 3). More of the policies that we monitored in this category involved expanding access rather than enacting mandates; for example, while the overwhelming majority of states enacted policies stating that the DOC would provide masks to residents and staff, less released policies stating that staff were *required* to wear masks and very few released policies *requiring* residents to wear masks. It should be noted that interim guidance provided by the CDC in March 2020 recommended face masks only for incarcerated persons who were confirmed or suspected of having COVID-19. Policies that increased access to hand sanitizer were also rarely reported, and some policies explicitly stated that sanitizer was still considered contraband.

**Table 3:** Prevention policies

***Policy Description***	***Frequency***		***Policy Type***		***Policy Form***		***Who Policy Primarily Affects***		
	*N*	%	*DOC-specific*	*Public health*	*Expand access*	*Mandate change*	*Resident*	*Staff*	*System*
Staff masks provided by DOC^a^	46	92%		X	X			X	
Resident masks provided by DOC^a^	46	92%		X	X		X		
Increased facility cleaning	46	92%		X		X			X
Staff required to wear masks^a^	38	76%		X		X		X	
Extra cleaning products provided to residents at no cost	32	64%		X	X		X		
Staff self-quarantine 14 days after positive	21	42%		X		X		X	
Residents must always wear masks^a^	21	24%		X		X	X		
Residents have the option to wear masks but not required^a^	21	42%		X	X		X		
Staff have option to wear masks	17	34%		X	X			X	
Sanitizer is available in limited locations	17	34%		X	X				X
Sanitizer is made widely available	15	30%		X	X				X
**% of policies monitored of total in category**			0%	100%	63.6%	36.4%	36.4%	36.4%	27.3%

^a^Note: No policies were mutually exclusive and percentage values indicate whether a policy has ever been released. Thus, policies that may seem as though they should total to 100% (e.g., staff masking optional and staff masking required) will not. Policies changed over time and therefore prison systems could have had both policies in place at some point during the pandemic

There were a range of other policies that were reported extremely infrequently including installing infrared cameras for temperature checks at facility entrances (*n = 2;* 4%). Notably, while incarcerated people were reportedly tasked with mask production in many prison systems (*n* = 38; 76%), very few DOCs reported policies that stated that incarcerated people would be paid for this work (*n* = 6; 12%).

#### Case identification and intervention policies

Policies included in this category were those that focused on identifying and responding to positive COVID-19 cases among prison staff and incarcerated persons (Table 4). These policies tended to involve the imposition of mandates; however, there were an array of policies that focused on expanding access to testing and medical services. About two-thirds of the policies that we monitored primarily affected residents, though policies affecting staff were common as well. For example, nearly all prison systems released policies indicating that staff would be verbally screened on-site and most also reported conducting staff temperature checks. Most states released policies indicating that incarcerated people who were identified as COVID-19 positive would be medically isolated. COVID-19 screening for new resident intakes and transfers was also common policy. Testing policies were much more variable. Less than 5% of prison systems had policies that explicitly stated that testing would be available to anyone in the facility upon request. Staff in some prison systems were subject to required COVID testing procedures, while other prison systems conducted no testing internally and instead indicated that staff testing should occur via an outside medical provider.

**Table 4:** Case identification and intervention policies

***Policy Description***	***Frequency***		***Policy Type***		***Policy Form***		***Who Policy Primarily Affects***		
	*N*	%	*DOC-specific*	*Public health*	*Expand access*	*Mandate change*	*Resident*	*Staff*	*System*
Staff verbal symptom screening occurring on site	48	96%		X		X		X	
Staff temperature checks occurring on site	44	88%		X		X		X	
Residents moved to medical isolation when COVID+	43	86%		X		X	X		
Testing available to residents who are symptomatic	41	82%		X	X		X		
New resident intakes/transfers are screened	40	80%	X			X	X		
Residents being mass tested at any point^a^	36	72%	X			X	X		
Residents moved to medical isolation if they were in contact with someone who tested COVID+	30	60%		X		X	X		
Residents moved to medical isolation when COVID test results are pending	29	58%		X		X	X		
Suspended resident medical co-pays for COVID-19 or related symptoms	20	40%	X		X		X		
Staff testing required at facility	17	34%		X		X		X	
Staff testing available but not required on-site	12	24%		X	X			X	
Suspended resident medical co-pays fully	11	22%	X		X		X		
Staff testing optional through private health care provider	6	12%		X	X			X	
Testing available to anyone in the facility upon request	2	4%		X	X		X	X	
**% of policies monitored of total in category**			28.6%	71.4%	42.9%	57.1%	64.3%	42.9%	0%

^a^Note: No policies were mutually exclusive and percentage values indicate whether a policy has ever been released. Thus, policies that may seem as though they should total to 100% (e.g., staff masking optional and staff masking required) will not. Policies changed over time and therefore prison systems could have had both policies in place at some point during the pandemic

#### Movement policies

Movement restriction policies, those that focused on reducing movements within and between carceral facilities, were one policy category that stood out as unique to DOCs during the pandemic. Policies stopping or restricting movement in some way were common—over half of prison systems released policies that indicated they would be: 1) restricting movements within their facilities, 2) requiring that new resident admits/transfers be quarantined, 3) implementing expedited resident releases, and/or 4) partially suspending resident transfers. However, few prison systems released policies indicating that transfers would be fully suspended. All policies that were monitored in this category were classified by our team as involving mandates and primarily affecting system operations. Overall, policies that affected movement within facilities were more common than policies that affected movement into or between facilities (Table 5).

**Table 5:** Movement policies

***Policy Description***	***Frequency***		***Policy Type***		***Policy Form***		***Who Policy Primarily Affects***		
	*N*	%	*DOC-specific*	*Public health*	*Expand access*	*Mandate change*	*Resident*	*Staff*	*System*
Movement restrictions within the facility	38	76%	X			X			X
Mandatory quarantine for new resident admits/transfers	33	66%	X			X			X
Resident transfers partially suspended (e.g., still transferring for medical/security reasons)	27	54%	X			X			X
Implemented expedited resident releases	27	54%	X			X			X
New resident intakes fully suspended	16	32%	X			X			X
System-wide quarantine	14	28%	X			X			X
Resident transfer volume reduced	11	22%	X			X			X
New resident intakes reduced by volume	11	22%	X			X			X
New resident intakes partially suspended (still transfer for medical/security reasons, etc.)	10	20%	X			X			X
Resident transfers fully suspended	10	20%	X			X			X
**% of policies monitored of total in category**			100%	0%	0%	100%	0%	0%	100%

#### Social communication and connection policies

Policies related to social communication and connection were also common, and fully DOC-specific; in short, this policy area suspended access to in-person visitation while temporarily increasing access to methods that could be used for remote social communication and connection. In-person visitation was suspended by all prison systems—notably this was the only policy reportedly enacted in all 50 states. Most prison systems also enacted policies that aimed to increase communication, such as by expanding access to phone, video, and/or messaging systems (Table 6). Free phone calls were by far the most common, though over half of prison systems had policies allowing free video calls as well. Our team classified all policies in this category as primarily affecting incarcerated people, as it was the resident population that would primarily bear the consequences of abrupt loss of and/or limitations to their access to their support networks.

**Table 6:** Social communication and connection policies

***Policy Description***	***Frequency***		***Policy Type***		***Policy Form***		***Who Policy Primarily Affects***		
	*N*	%	*DOC-specific*	*Public health*	*Expand access*	*Reduce access*	*Resident*	*Staff*	*System*
In-person visitation is suspended	50	100%	X			X	X		
Free phone calls	45	90%	X		X		X		
Free video calls	29	58%	X		X		X		
In-person visitation is restricted	14	28%	X			X	X		
Video calls other (e.g., made available if not previously provided)	10	20%	X		X		X		
Phone calls other	6	12%	X		X		X		
Reduced fee video calls	5	10%	X		X		X		
Reduced fee phone calls	4	8%	X		X		X		
Longer or more phone calls	3	6%	X		X		X		
Longer or more video calls	2	4%	X		X		X		
**% of policies monitored of total in category**			100%	0%	80%	20%	100%	0%	0%

#### Access to programming, recreation, and privileges

This category includes policies that are related to aspects of prison life that are typically considered “privileges” by DOCs. The vast majority of policies in this category reflected *restrictions* including on resident programming, jobs/work release, and recreational time (Table 7). Some policies describing programming restrictions explicitly acknowledged potentially problematic consequences of such restrictions, including those that could delay their release (e.g., residents being unable to complete required programming on as quick of a timeline).

**Table 7:** Programming, recreation, and privilege policies

***Policy Description***	***Frequency***		***Policy Type***		***Policy Form***		***Who Policy Primarily Affects***		
	*N*	%	*DOC-specific*	*Public health*	*Expand access*	*Reduce access*	*Resident*	*Staff*	*System*
Programming restricted in some way	48	96%	X			X	X		
Work release jobs limited	16	32%	X			X	X		
Expanded privileges (e.g., more snack line time)	11	22%	X		X		X		
Reduced privileges (e.g., day passes, furloughs)	9	18%	X			X	X		
Recreation/yard time increased	4	8%	X		X		X		
Recreation/yard time decreased	4	8%	X			X	X		
Recreation/yard time suspended	2	4%	X			X	X		
**% of policies monitored of total in category**			100%	0%	28.6%	71.4%	100%	0%	0%

## Discussion

People in prison are fully reliant on these facilities and their staff for their health needs. Thus, the health and safety of people who have been and who are currently incarcerated during the COVID-19 pandemic demanded rapid, health-focused responses by systems that were not built to promote health and that often have a substantial shortage of healthcare providers and are notorious for poor quality care. Even as state-and national-level guidance on reducing virus transmission was being determined, prisons were having to decide and implement pandemic responses in the hopes of preventing outbreaks or, at least, limiting them. The full outcome of these efforts remains to be seen as the pandemic remains ongoing. Our work provides foundational knowledge by describing the wide breadth of policies that were reported in the first year of the pandemic. This information may be used as a base for quantitative work on policy effectiveness and as well as qualitative studies examining policy implementation.

Our results—which highlight the social, privilege, and programming restrictions that incarcerated people experienced during the pandemic—underscore the importance of examining policy effects through the lens of both possible benefits and possible harms. We would hypothesize that some policies could have had a decidedly negative effect because the very policies that will stop the spread of COVID-19 are likely to worsen other aspects of health and further limit already tenuous access to health care services and enrichment programs. For example, programming is typically offered in prisons, including but not limited to education, substance abuse and mental health treatment, and religious services. Community volunteers and contracted providers normally provide a portion of this programming (Taxman et al., 2007). Thus, limitations on facility access have the collateral consequence of limiting programming. Additionally, many facilities enacted medical isolation procedures, requiring people who are incarcerated to stay isolated within single cells or barracks. Given that most prison-based programming is provided in groups, such policies effectively suspend access to these programs indefinitely. Ironically, in some states that are moving to decarcerate, a lack of access to treatment services is the very thing that is preventing early releases because people are unable to meet the conditions required to be eligible (Widra & Sawyer, 2020).

Movement and social restriction policies (e.g., lockdowns, visitation restrictions, suspension of yard/rec time and other social activities) are also of concern because of the negative impact of isolation. Indeed, research has found that people who are subjected to full isolation while incarcerated have higher rates of death by suicide, homicide, and opioid overdose post-release (Brinkley-Rubinstein et al., 2019). Thus, the precautions that are necessary to limit outbreaks may ultimately cause increased risk of future morbidity and mortality. Efforts to reduce the likelihood of such negative outcomes are in place in some states (e.g., greater access to phone, email, and video visitation), but these substitutes are clearly not the same as in-person visitation.

It is impossible to know whether the extreme infection rates in prisons are due to the policies being overall ineffective or implemented poorly. The substantial volume of lawsuits currently facing state prison systems (e.g., *Valentine v. Collier, Waddell v. Taylor)* highlight the rampant problems in policy implementation that could reduce or eliminate effectiveness of even the most robust policies. While the CDC has published ongoing guidelines for U.S. correctional facilities, legal scholars have argued that courts are giving excessive deference to CDC guidance given its informal nature (Conditions of Confinement, COVID-19, and the CDC, 2021) and other researchers have argued that collaboration between state department of corrections and public health are necessary to better address COVID-19 among incarcerated people (Hamblett et al., 2022). CDC guidance falls short of recommendations by international and national agencies which called for safe decarceration (e.g., Human Rights Watch (2020); National Academies of Sciences (2020)). Nevertheless, understanding the degree to which pandemic procedures were or were not implemented in the manner dictated by policy has far-reaching health implications for those living in prisons and jails, those who work in these facilities, and the communities which surround them. A full accounting of institutional pandemic response is also vital in crafting policies designed to lessen the effects of the next pandemic and other disease outbreaks. Taken together with the steady drumbeat of new lawsuits, Occupational Safety and Health Administration violation claims, and desperate pleas from prison staff and residents, these examples strongly suggest that there exist further cases of DOC policy non-compliance which will require study.

### Limitations

Our study was limited by the availability of information; we could only incorporate public-facing directives and it is possible that internal communication was used to disseminate some changes. We also monitored only public DOC sources (i.e., DOC webpages, Twitter accounts, Facebook pages, and public-facing statements) and it is possible that information released via other venues (e.g., internal staff emails) could have added to our accounting. This strategy was important for methodological rigor (i.e., standardizing the approach to sources that were included); however, for this reason, we anticipate that the results that we report are the *minimum* number of states that enacted each of the policies examined. Indeed, due to their direct work with state prison systems in other contexts, the authors are aware of policies that could not be counted in this study because they did not appear in public-facing documents but that were being implemented in practice at various times during the pandemic.

Even within public sources, there was great variability as to ease of access of information, and it is thus possible that despite our best efforts, documents that should have been included were missed. As above, we would assume that any errors in our accounting of policies released would lead to underestimates. Additionally, we have chosen to focus our analysis on summarizing policies that were ever in place. We do not view this as a major limitation because, once released, policies were rarely lifted during the time frame of the study. Visitation policies are a notable exception as some states did resume visitation. Finally, it is worth noting that policies were at times extremely vague (e.g., using terminology such as “no unnecessary transfers”). While we had internal protocols regarding how to handle these instances, it is possible that interpretations of terms like “unnecessary” varied widely and influenced implementation.

A potentially larger limitation to this study—and in many policy-focused research studies—is that we were unable to assess policy implementation. We had endeavored to do so when we first launched the project; however, it became clear that this would be impossible due to the vagueness that was written into many policies (e.g., requiring that masks be worn by staff and residents only in certain, ill-defined, situations; providing lengthy descriptions of when masks were and were not required). It was also notable that for many of the social policies that involved free access to communication, such as free phone calls or video visits, was the result of fee waivers by the vendors that provide those services. Thus, the degree to which residents were able to access such resources remains unclear and could have been counteracted by other policies (e.g., movement restrictions) that were in place simultaneously.

## Conclusion

This study provides a foundation from which to begin examining the impact of COVID-19 mitigation policies on the health and wellness of people who were incarcerated during the pandemic. As the most comprehensive overview of the policy landscape in prisons during the pandemic—and only one utilizing data from long-term web scraping—the study provides valuable descriptive knowledge on the frequency of policy implementation nationally. This information can be used to contextualize future studies on the long-term effects of the pandemic on individual and community health as our data summarizes the degree to which many policies were universally enacted in prisons versus being more variable or rare.

Our study also provides data that can be used to inform attempts to mitigate collateral consequences of the pandemic on incarcerated people. For example, our data demonstrates that suspension of in-person visitation was the only policy that was universally enacted—something that cannot be said for masking of incarcerated people or carceral staff. Given the pivotal role of family and social connection on health outcomes, this knowledge suggests a need for efforts to 1) examine how policy can be used to expedite releases from prisons to minimize harm during the pandemic and 2) increase the robustness of efforts to offset potential harms beyond the slight expansions in distance-communication privileges that were seen in many states.

Research that empirically examines the short- and longer-term impact of policies on the health of people who are incarcerated is also sorely needed. While it is tempting to assume that policies that were intended to mitigate COVID-19 transmission helped, this is an assumption in need of empirical testing and critical examination. Indeed, it is possible that some policies (e.g., policies that increased isolation or restricted access to therapeutic programming) also created collateral harms that remain to be seen. It is also possible that some policies that are thought to be effective in community settings had minimal or no impact on health because of the structural characteristics of carceral environments (e.g., building features making social distancing impossible despite policies encouraging it). Future research should interrogate the effects of the full range of policies that were enacted to inform the development of and advanced planning for policies and procedures for additional infectious disease outbreaks in these settings.

## Data Availability

Not applicable.

## Appendix 1

Table 8

**Table 8:** COVID prison project policy codebook

***Codebook variable and definition***	
Code	Definition
Staff masks provided by DOC^a^	PPE provided by employer/DOC
Resident masks provided by DOC^a^	Resident masks are provided by DOC or facility.
Increased facility cleaning	Facility is cleaning more frequently.
Staff required to wear masks^a^	Staff are required to wear PPE/Masks while onsite
Extra cleaning products provided to residents at no cost	Facility has more hygiene products available and provides them for free.
Staff self-quarantine 14 days after positive	Required quarantine for 14 days after a staff person self-reports or tests positive.
Residents must always wear masks^a^	Facility mandates that everyone who is incarcerated is required to wear masks.
Staff have the option to wear masks	Staff have the option to wear PPE/Masks while onsite
Residents have the option to wear masks but not required^a^	Residents have the option to wear PPE/Masks
Sanitizer is made widely available	Facility has made sanitizer widely available (for example, distributing it to individual people)
Sanitizer is available in limited locations	Facility is placing sanitizer in strategic locations (entrances, cafeterias)
Staff temperature checks occurring on site	Having temperature read on-site at facility
Staff verbal symptom screening occurring on site	Staff self-report of symptoms conducted at facility
Residents moved to medical isolation when COVID+	Residents go into quarantine after a positive test.
New resident intakes/transfers are screened	New screenings practices for those coming into the facility at intake or via transfer. Ex. questionnaires, taking temperatures, etc.
Testing available to residents who are symptomatic	Tests were made available to anyone who indicated they were showing COVID-19 symptoms
Residents being mass tested at any point^a^	Mass testing for everyone who is incarcerated within the facility regardless of symptomology.
Residents moved to medical isolation if they were in contact with someone who tested COVID+	Residents go into quarantine after coming into contact with someone who has tested positive.
Residents moved to medical isolation when COVID test results are pending	Residents go into quarantine during a pending test.
Staff testing required at facility	State reports requiring staff to be tested
Suspended resident medical co-pays for COVID-19 or related symptoms	Facility suspends co-pays fully for COVID/possibly-related symptoms expenses.
Staff testing available but not required on-site	State reports that testing is available on-site to staff as needed
Suspended resident medical co-pays fully	Facility suspends co-pays fully for all medical expenses.
Staff testing optional through a private health care provider	State reports that testing is done for staff with the staff’s private health care provider
Testing available to anyone in the facility upon request	Testing available to anyone in the facility upon request
Movement restrictions within the facility	Individuals are limited in their ability to move (ex. housing pods).
Mandatory quarantine for new admits/transfers	New intake or transfer have a required quarantine period upon admittance (e.g., 7 days, 14 days, until negative test).
Transfers partially suspended (e.g., still transferring for medical/security reasons)	Some individuals are transferred the facility (medical, security reasons)
Implemented expedited releases	Facility is releasing individuals early (this could be for one subgroup of the population or restricted).
System-wide quarantine	Full facility goes on lockdown due to COVID-19 diagnosis present
New intakes fully suspended	No new individuals are entering the facility.
Transfer volume reduced	Reduction of number of transfers
New intakes partially suspended (still transfer for medical/security reasons, etc.)	Some individuals are entering the facility (medical, security reasons)
New intakes reduced by volume	Reduction of the number of intakes.
Transfers fully suspended	No new transfers.
In-person visitation is suspended	In-person visitation is completely suspended.
Free phone calls	Facility provides free phone calls to compensate for visitation restrictions.
Free video calls	Facility provides free video calls to compensate for visitation restrictions.
In-person visitation is restricted	In-person visitation is more limited than regular procedures.
Video calls other (e.g., made available if not previously program)	Other practices concerning video calls.
Phone calls other	Other practices concerning phone calls.
Reduced fee video calls	Facility reduces fees for video calls to compensate for visitation restrictions.
Reduced fee phone calls	Facility reduces fees for phone calls to compensate for visitation restrictions.
Longer or more phone calls	Facility provides more time for phone calls to compensate for visitation restrictions.
Longer or more video calls	Facility provides more time for video calls to compensate for visitation restrictions.
Programming restricted in some way	Programming done inside prisons that are mediated by paid internal staff providers are modified due to COVID (ex., suspended, operating in but in less frequency, done remotely or in reduced size)
Work release jobs limited	Facility cuts back on the number/type of work release jobs but does not fully suspend.
Expanded privileges (e.g., more snack line time)	Increase in the extent of privileges given to residents (e.g., having longer time to be in the snack line, free movie/game access, longer furloughs).
Reduced privileges (e.g., day passes, furloughs)	Decrease/reduction in the privileges given to residents normally (e.g., day passes or furloughs given).
Recreation/yard time increased	Increase in the amount of time residents are allowed to be in the yard/rec.
Recreation/yard time decreased	Decrease in the amount of time residents are allowed to be in the yard/rec.
Recreation/yard time suspended	Residents no longer able to have rec/yard time because of this COVID pandemic.

^a^Note: No policies were mutually exclusive and percentage values indicate whether a policy has ever been released. Thus, policies that may seem as though they should total to 100% (e.g., staff masking optional and staff masking required) will not. Policies changed over time and therefore prison systems could have had both policies in place at some point during the pandemic

## Appendix 2

Table 9

**Table 9:** Communication methods by state prison systems

State	Facebook	Twitter	DOC Webpage	State	Facebook	Twitter	DOC Webpage
AK	1	0	1	MT	1	0	1
AL	1	0	1	NC	1	1	1
AR	1	1	1	ND	1	0	1
AZ	0	1	1	NE	1	0	1
CA	1	1	1	NH	1	1	1
CO	0	1	1	NJ	1	1	1
CT	0	1	1	NM	0	0	1
DE	1	1	1	NV	1	1	1
FL	1	1	1	NY	0	1	1
GA	1	1	1	OH	1	1	1
HI	1	1	1	OK	1	1	1
IA	1	1	1	OR	1	1	1
ID	1	1	1	PA	1	1	1
IL	1	1	1	RI	1	1	1
IN	1	1	1	SC	1	1	1
KS	1	1	1	SD	0	0	1
KY	1	0	1	TN	1	1	1
LA	1	1	1	TX	1	0	1
MA	1	0	1	UT	1	0	1
MD	1	0	1	VA	1	1	1
ME	1	0	1	VT	1	1	1
MI	1	1	1	WA	1	1	1
MN	1	1	1	WI	1	1	1
MO	1	1	1	WV	1	0	1
MS	1	1	1	WY	0	0	1

Note: A value of "1" indicates that the communication method was recorded as being used to release relevant information atleast once during the study period. A value of "0" indicates that the communication method was not used

## Footnotes

1 This approach ensured that our policy coding was done at a granular level and that the data that we present is reflective of specific, non-overlapping policies. In other words, while the broader conceptual categories outlined in Table 1 may theoretically overlap (e.g., restrictions on movement would theoretically overlap with changes in the social and privilege categories), our frequency results (i.e., those reported in Tables 3, 4, 5, 6 and 7) are focused at the level of individual, non-overlapping policies.

2 We focused on whether each state *ever* enacted each of our policies of interest because, over time, we learned that dates of policy onset and offset were not consistently or clearly reported in a way that would allow for more nuanced longitudinal reporting while still maintaining rigor. The dates that policies were intended to end were especially murky. We also found that the source data would at times refer to things that a state DOC had already enacted; thus, in these cases, unless a specific onset date was specified by the data source we would not have information on when the policy/practice had started—just that it was currently in place.

3 For nine states, there was only a single post.
